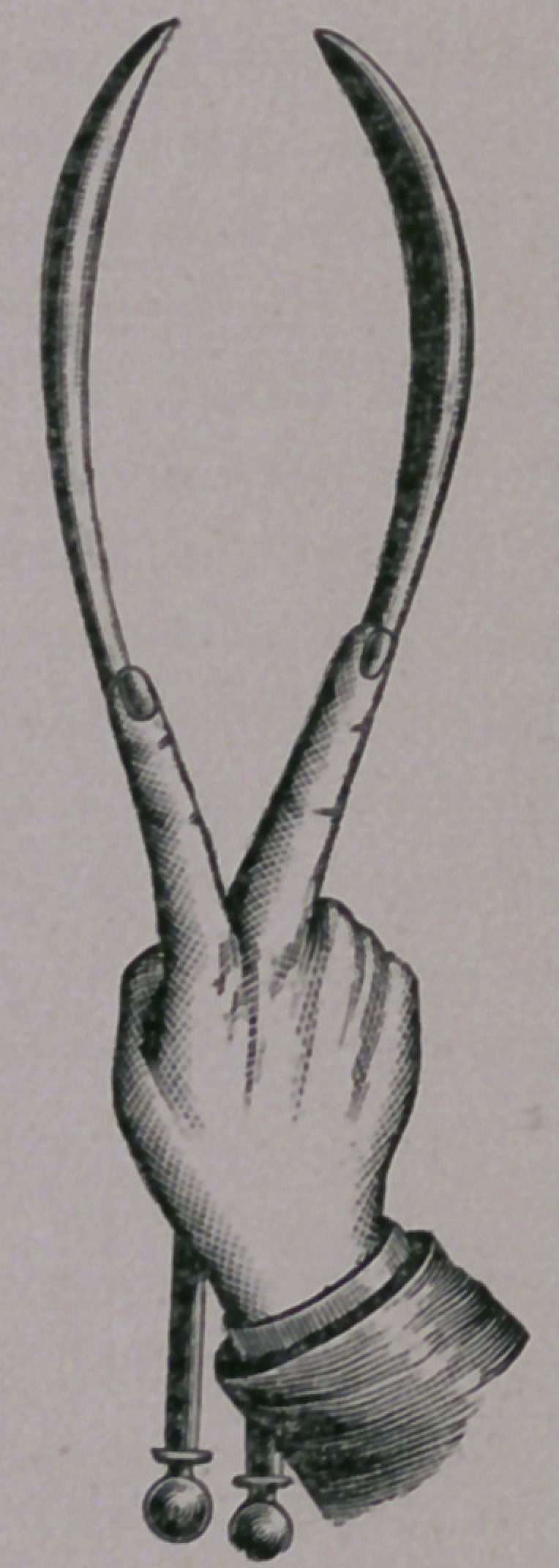# How to Use the Forceps

**Published:** 1894-05

**Authors:** 


How to Use the Forceps, with an introductory account of the Female Pelvis and of the Mechanism of Delivery. By Henry G. Landis, A. M., M. D., Professor of Obstetrics and Diseases of Women and Children in Starling Medical College, Columbus, Ohio. Revised and enlarged by Charles H. Bushong, M. D. New York: E. B. Treat, Publisher, 5 Cooper Union. 1894. Price, $1.75.

This valuable monograph of about two hundred pages is the most complete, minute, and painstaking exposition of this subject the writer of this notice has ever seen.

It comprises essentially three parts: The mechanism of labor, the forceps, and the application and cases.

A thorough analysis of the female pelvis in especial relation to its function as a channel for the fœtal head is given, with an abundance of diagrams to enable the attentive reader to acquire a clear mental picture of the whole tract, and of the position of the head at every moment of its progress. This section alone is admirable and must commend the book. But when it dawns upon the reader that it is only the preparation for a clear understanding of the proper use of the forceps without the excessive traction which seems to the humane mind so horrible, and that this minute anatomy prepares the reader's mind so that he shall be perfectly able to agree with the author in his denunciation of brute force in the application of the instruments, and perfectly prepared to adopt the author's improved method, then the book is seen to be a very important benefaction to mankind and aid to the alleviation of the sufferings of child-birth.

It will be necessary to quote a couple of pages to make our point clear:

Page 121: "The forceps having been applied, the next question is, what are we to do with them? Are we to pull the head out by direct traction or to pry it out by leverage, and shall it be compressed during either of these movements?

"The following propositions may be laid down as a starting point: *Firstly.*—If the Davis forceps (or any other having a sufficient pelvic and head curve) are applied to the sides of a head at the inlet in the first vertex position, the general line of the blades will be parallel to the axis of the presenting plane of the head. *Secondly.*—If traction is made in the line of the blades, the distal end of the blades will press upon the head, and if the latter is movable will push it onward in the line of the axis of the presenting plane. *Thirdly.*—If during traction the line of the blades is kept parallel with the axis of the canal in which the head is placed, the axis of the presenting plane of the head will be kept in coincidence with the axis of the canal in which it moves.

"This is what takes place in normal labor, and this is what it should be our aim to imitate with the forceps. It ought not to require a mathematical demonstration to show that when the head is kept in this exact relation with the pelvic canal it will move with the least possible expenditure of force. If instead of this the force be so directed as to push or pull it alternately against the sides of the pelvis, more force will be required, unless the laws of mechanics are altered for the benefit of obstetricians. And yet the great majority of obsteric writers recommend that traction be supplemented by leverage, and that the handles of the forceps should be swayed from side to side that the head may be pried out as well as pulled out of the pelvis. From this it may be inferred, however presumptuous the inference may seem, that they do not make traction in the right direction."

Page 127: "The method which seems to be the correct one I will now attempt to describe. When the forceps are applied at the inlet the handles are seized by the right hand from above and held firmly, compressing the head as little as possible at first. The left hand is placed so that the ball of the thumb comes over the lock (see Figure), while the index finger rests upon the upper arm of one blade and the middle finger upon the other. Now, while the right hand holds the handles almost at rest, the fingers of the left *push* upon the blades so as to move them and the contained head downwards, backwards, and a little to the left of the median line. Secondly, while the fingers are pushing downwards in this way, we may also make use of them as a fulcrum, and by elevating the handles cause the blades to move in an opposite manner, but care must be taken that the force thus applied by the right hand is not enough to overbalance the downward pressure of the left, else we will merely extend the head without propelling it. It is sometimes convenient to vary the position of the left hand and fingers, but the principle is the same, that pushing and not pulling is the first step in traction. When the head begins to descend we may place three fingers between the blades, the thumb and the little finger being upon the outside, and combine a pulling with a pushing motion upon the blades. But throughout the handles are simply elevated and not pulled upon, or but slightly, having due regard to the proper direction, and bringing them into the median line only when the head has reached in inferior strait. When the head is delivered the handles will lie upon the abdomen of the mother. This, in brief, is the method which I employ and advise."

The introduction of these two long extracts has a two-fold purpose: to draw the attention of the profession to the important character of this book, and to give to those readers who may not have time to devote to the book and who do not make a specialty of obstetrics a clear conception of the latest, most enlightened, and most humane way of applying the forceps. It is advisable, however, to study carefully every page of the book itself to acquire the idea perfectly.

## Figures and Tables

**Figure f1:**